# White Mulberry Plant Extracts in Cardiovascular Prevention: An Update

**DOI:** 10.3390/nu17142262

**Published:** 2025-07-09

**Authors:** Valentina Trimarco, Paola Gallo, Seyedali Ghazihosseini, Alessia Izzo, Paola Ida Rozza, Alessandra Spinelli, Stefano Cristiano, Carlo De Rosa, Felicia Rozza, Carmine Morisco

**Affiliations:** 1 Department of Neuroscience, Reproductive Sciences, and Dentistry, Federico II University, 80131 Naples, Italy; valentina.trimarco@unina.it; 2 Department of Advanced Biomedical Sciences, Federico II University, 80131 Naples, Italy; paola.gallo@unina.it (P.G.); ali_ghz_95@yahoo.com (S.G.); alessandra.spinelli@unina.it (A.S.); stefano.cristiano@unina.it (S.C.); 3 International Translational Research and Medical Education (ITME) Consortium, Academic Research Unit, 80100 Naples, Italy; alessiaizzo45@gmail.com (A.I.); paolarozza21@gmail.com (P.I.R.); feliciarozza@libero.it (F.R.); 4 Department of Medicine, Surgery and Dentistry, University of Salerno, 84081 Salerno, Italy; derosa.medicolegale@gmail.com

**Keywords:** dietary supplements, nutraceuticals, oxidative stress, diabetes, *Morus alba*, obesity

## Abstract

This review examines the principal preclinical and clinical findings assessing the effects of White Mulberry (*Morus Alba Linn*) plant extract supplementation currently available. Since it is one of the most cultivated species of mulberry tree, it has caught the eye of researchers for its rich phytochemical profile as well as multi-purpose usages. The leaves, fruits, and other parts of the White Mulberry plant take on the role of valuable sources of bioactive compounds, including flavonoids, phenolic acids, terpenoids, and alkaloids. These secondary metabolites have a wide range of health benefits, such as antioxidant, anti-inflammatory, and antidiabetic properties. Commonly used as dietary supplements, White Mulberry plant extracts have shown their great capacity in improving metabolic profile, decreasing the cardiovascular risk, and supporting overall health.

## 1. Introduction

Herbal agents and natural compounds have been drawing increasing focus in many countries [1,2]. Several studies have demonstrated that consuming various plants can help decrease the plasma levels of glucose and lipids while strengthening the body’s antioxidant defense system [3]. Thus, many bioactive food components have been evaluated for their potential to prevent different chronic and degenerative diseases. Moreover, bioactive compounds in fruits and vegetables have attracted interest for their antioxidant properties and benefits in the management of obesity and metabolic disorders [4]. Among these natural products, mulberry—from the genus Morus in the Moraceae family—has been widely cultivated in countries such as China, Korea, Japan, and Taiwan [5].

Medicinal plants from the Moraceae family are known for their wide range of applications in fields such as cosmetics, agriculture, and pharmaceuticals. A notable example is the White Mulberry, also known as *Morus Alba Linn*. Native to China, this species is now grown worldwide, largely due to its role in sericulture [6].

This review examines the pharmacologic activities and principal preclinical and clinical findings assessing the effects of White Mulberry plant extract supplementation currently available. For this purpose, an electronic search of the available literature was performed using the following databases: EMBASE, NCBI, and PubMed. The key words used for the search were: “White Mulberry” and “*Morus alba* L.” associated with “active compound”, “pharmacological activities”, “oxidative stress”, “diabetes”, “obesity”, “metabolism”, “adiposity”, “animal studies”, “clinical trials”, “cardiovascular risk”. For this review, only papers in English were considered, and papers not strictly related to the aim of the review were excluded. Out of the 432 papers considered relevant for this review, we included 96 articles.

Traditionally, all parts of the mulberry plant have been used to help various physiological conditions, including diuresis, sedation, cooling the body, improving tone, and treating neuropathy [7]. In particular, its leaves have been used as cooling agents, sweat inducers, and antipyretics, while also being recognized for their nutritional value, as they are rich in proteins and polysaccharides [8]. The principal bioactive elements found in mulberry leaves include amino acids, flavonoids, polysaccharides, steroids, and vitamins, all of which contribute to treating several internal diseases and infections. Compared with Red Mulberry and Black Mulberry, White Mulberry as received more scientific interest for many reasons. While White Mulberry originated in China, it has now been widely farmed and naturalized across Asia, Europe, and North America. Its broad availability has provided researchers a large amount of plant material and study samples, encouraging more extensive research into its properties.

In Chinese medicine, the White Mulberry has been used to treat fever, to protect the liver, to improve eyesight, strengthen joints, to stimulate diuresis, and to decrease arterial blood pressure [9]. In Korea and Japan, mulberry leaves are used by diabetic patients to ameliorate glycemic control. Additionally, mulberry leaves are effective in relieving hangovers from alcohol [10].

Being rich in metabolites such as alkaloids, flavonoids, and phenolic compounds makes White Mulberry an important curative resource. Due to possessing these compounds, it plays a role in a variety of biological activities, including antioxidant, anti-inflammatory, and neuroprotective effects. Furthermore, White Mulberry has shown interesting prospects in preventive medicine, for example, in the treatment of type 2 diabetes, overweight, and neurodegenerative diseases.

Nowadays, there is convincing evidence for the use of White Mulberry, documenting a strong rationale for further investigations. To date, 1107 relevant papers have been registered, along with over 24,000 citations. The scientific knowledge about mulberry flavonoids has grown significantly [11,12], with current research focusing mainly on the phytochemistry of mulberry and its biological properties, particularly its antioxidant potential.

## 2. Phytochemistry

The chemical analysis of White Mulberry leaves has identified a variety of substances, including alkaloids, amino acids, β-carotene, cudraflavone B, isoquercitin, moracetin, prenylflavone, organic acids, quercetin-3-triglucoside, rutin, tannic acid, phosphorus, calcium, magnesium, iron, coumarins, vitamin C, dihydroxycoumarin, oxyresveratrol, protein, and zinc [13].

## 3. Flavonoids

Mulberry plants are well known for their rich flavonoid content. It is well known that mulberry leaves contain kaempferol, kuwanons, quercetin, moracin flavans, moragols, and morkkotins [14]. Rutin, kaempferol, and astragalin have been detected in concentrations ranging from 4.34 mg/g to 0.53 mg/g of total content [15]. Other flavonoids, such as flavonols, glycosides, quercetin-3-(6-malonylglucoside), and isoquercitrin, are also extracted from the mulberry plant. In addition to their nutritional benefits, these flavonoids also exert a protective action against allergic reactions and viral infections [16].

## 4. Alkaloids

Twenty-one alkaloids have been isolated from mulberry leaves. The most relevant include D-fagomine (FAG), isofagomine, cis-5-hydroxypipecolic acid, 4-*O*-β-D-glucopyranosyl-fagomine (Glu-FAG), 1-deoxynojirimycin (1-DNJ), 2-*O*-α-D aurantiamide acetate, galactopyranosyl-deoxynojirimycin (GAL-DNJ), trans-5-hydroxypipecolic acid, methylpyrrolidine carboxylic acid, and pipecolic acid. Among these, 1-deoxynojirimycin (1-DNJ) is considered the most active substance and has been investigated by many researchers for its effective and specific capability to inhibit several enzymes involved in several biochemical pathways, such as glycoprotein sugar chain maturation and intestinal digestion. 1-DNJ also acts as an antidiabetic agent by decreasing the rate of carbohydrate breakdown into monosaccharides, and it can also interfere with the intestinal glucose absorption, thereby reducing postprandial plasma glucose levels [17].

## 5. Anthocyanins

Mulberry leaves provide nutritional benefits, such as minerals, anthocyanins, phytonutrients, and vitamins. Anthocyanins are especially beneficial for health due to their antioxidant and anti-inflammatory properties. They also have a strong ability to interfere with the oxidation of lipids and have anti-metastasis activity [18,19].

## 6. Polysaccharides

Polysaccharides in mulberry leaves have gained attention from researchers for their interesting biological properties. They stimulate the insulin expression and regulate the metabolism of glucose in the liver [20]. In mulberry leaves, polysaccharides are mostly found in the inner epidermal cells [21]. These polysaccharides are made up of various monosaccharides like arabinose, fructose, galactose, galacturonic acid, glucose, mannose, xylose, rhamnose, galacturonic acid, glucuronic acid, and sorbose [22].

## 7. Amino Acids

Amino acids are essential for the synthesis of antioxidant enzymes. Moreover, some amino acids can directly scavenge oxygen free radicals. The four main amino acids present in mulberry leaves are asparagine, alanine, proline, and gamma-aminobutyric acid ( GABA) [23].

**Figure 1:**
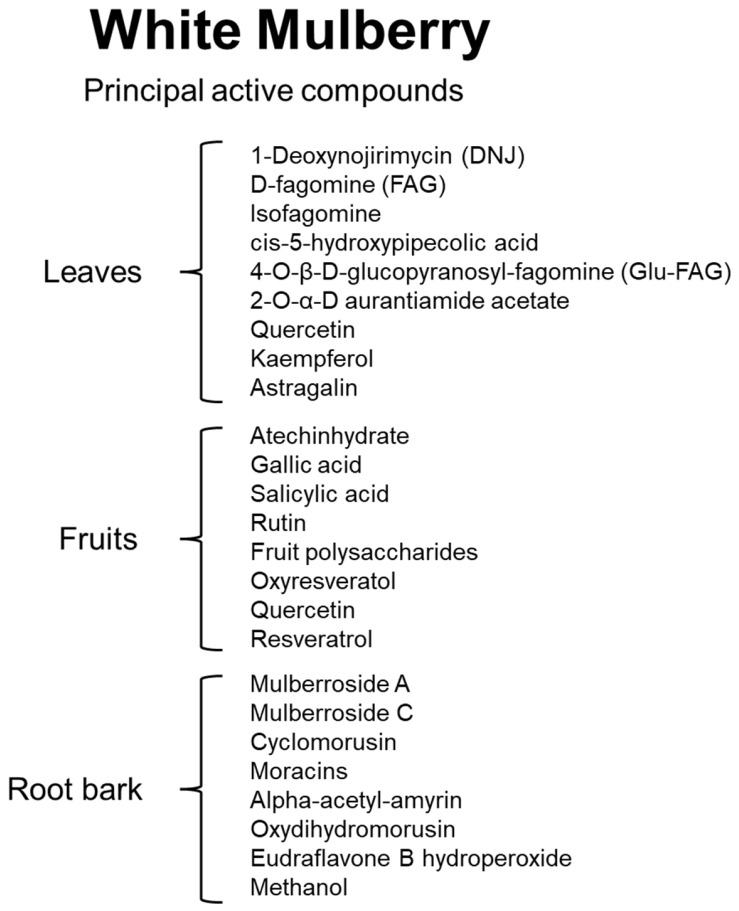
Principal active compounds present in the leaves, fruits, and root bark of White Mulberry.

**Figure 2:**
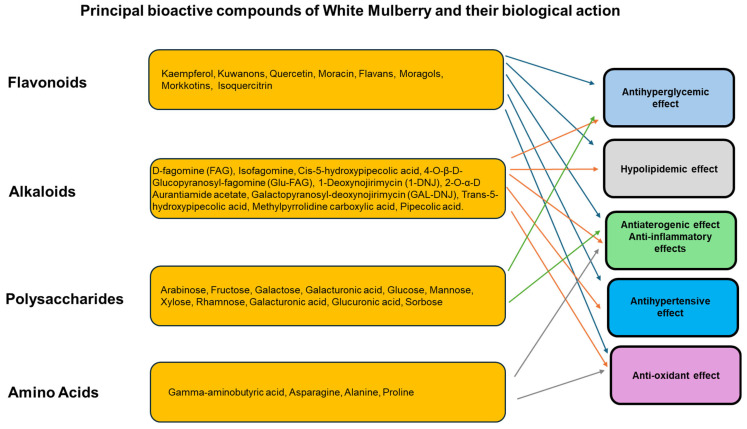
Principal bioactive compounds present in the White Mulberry and their biological action.

Figure 1 shows the principal active compounds found in the leaves, in the fruits, and in the root bark of the White Mulberry. Figure 2 reports the principal bioactive compounds of the White Mulberry and their biological action.

## 8. Pharmacological Activities of Mulberry Leaves

### 8.1. Antioxidant Activity

Free radicals are reactive molecules. They are generated in cells as byproducts of metabolism [24]. It is important to note that plants have the natural characteristic to produce different antioxidative enzymes, like ascorbate, catalases, glutathione reductases, peroxidases, and polyphenol oxidases, especially during environmental stress. Three flavonoids—quercetin, rutin, and isoquercetin—are the principal antioxidant components present in the ethanol extracts of mulberry leaves [8]. In particular, the high concentration of quercetin in mulberry leaves contributes to reducing the oxidative stress [25]. For these specific properties, mulberry leaf extracts show cytotoxic effects against cancer cells. For example, galactose-bonded lectin isolated from mulberry leaves has documented cytotoxic activity against human breast cancer cells with an IC50 of 8.5 μg/mL, and against colon cancer cells with an IC50 of 16 μg/mL [26]. Many phenolic compounds present in mulberry leaves induce anticancer activity in hepatoma cells by arresting the cell cycle at the G2-M phase and inhibiting topoisomerase II activity. The flavonoids and quercetins in mulberry leaves have also shown selective cytotoxicity against human ovarian and gastric cancer cells [27]. In addition, the polyphenols and alkaloids found in mulberry leaves are known to enhance cognition and slow down neurodegeneration [28]. Experimental studies have documented that substances extracted from mulberry leaves can be used in neurogenesis for treating neurodegenerative processes, including Alzheimer’s disease. These extracts can prevent the aggregation of amyloid β-peptide (91–42) and reduce the neurotoxic effects caused by amyloid β-peptide (1–42) [29].

### 8.2. Anti-Metabolic Disorder Activity

A growing body of evidence has proven that mulberry leaves, through flavonoids, exert a relevant hypoglycemic action. Mulberry leaves contain 1-DNJ, which is able to inhibit the α-amylase and α-galactosidases [30]. The DNJ content in leaves from 132 varieties of nine Morus species has been determined [31], with 58 of these varieties belonging to White Mulberry. Interestingly, one oral hypoglycemic agent marketed in Europe is synthesized from 1-DNJ. The flavonoids and related compounds in mulberry leaves also show antidiabetic effects. Leaves from South American mulberry species were found to contain benzofuran derivatives, which showed antidiabetic activity in an animal model of diabetes [32]. Some purified flavone fractions from mulberry were found to activate α-glucosidase enzymes, which, in turn, increase blood glucose levels [33]. On the other hand, polysaccharides present in mulberry leaves have strong potential for inhibiting α-glucosidase. Moreover, mulberry fruit contains significant amounts of bioactive agents, including anthocyanins, flavonoids, phenolics, and other antioxidants, which may offer benefits against obesity, cholesterol, and protective effects for the liver [34]. The polyphenols are a complex chemical family, which can explain the complexity of their physicochemical properties and, consequently, the conflicting results among the different studies. It has been speculated that the observed health benefits evoked by mulberry leaves may not be due to a single compound but to the combined action of different compounds, including anthocyanins, flavonoids, and phenolics [35]. Additionally, freeze-dried mulberry fruit powder contains high levels of dietary fiber, vitamins, antioxidants, and linoleic acid, which may also contribute to its hypolipidemic activity [36].

### 8.3. Anti-Inflammatory Activity

The methanol root bark extract of White Mulberry has been reported to exert an anti-inflammatory effect [37]. This action is mediated through the inhibition of NF-κB and activation of the MAP kinase ERK1/2. Among the compounds isolated from the methanol root bark extract, kuwanons C and G have been identified as having anti-inflammatory activity. Similarly, the methanol branch extract of White Mulberry and its active compound oxyresveratrol also demonstrate anti-inflammatory properties [38]. The molecular mechanism that accounts for these effects involves inhibition of CXCR-4-mediated chemotaxis and interference with the MEK/ERK pathway in T cells and other immune cells.

## 9. Animal Studies

### 9.1. Antidiabetic Effects

**Figure 3:**
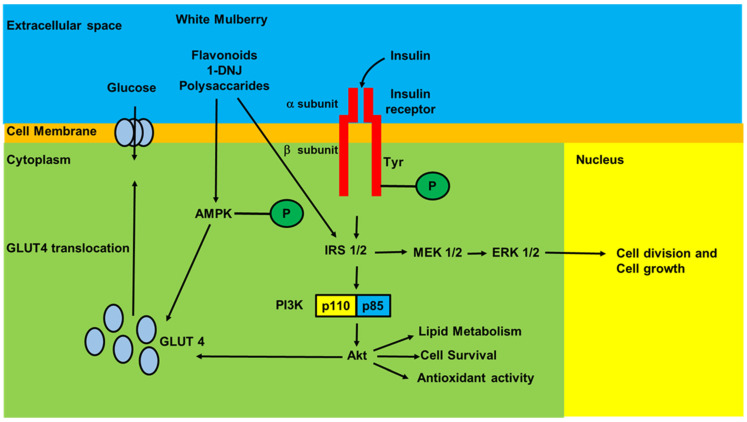
Schematic representation of early steps of insulin signaling, and the principal mechanisms that account for the effects of White Mulberry extracts on glucose homeostasis. Binding of insulin to the α sub-unit of its own receptor stimulates the auto-phosphorylation of tyrosine residues of β sub-unit, which, in turn, induces the tyrosine phosphorylation and activation of IR substrates (IRS), IRS-1 and IRS-2. The binding of phosphorylated IRS1/2 to the regulatory sub-unit p85 of phosphoinositide-3 kinase (PI3K) activates the catalytic sub-unit p110, which, in turns phosphorylates/activates the serine/threonine kinase Akt (called also PKB), which stimulates the glucose uptake through the translocation of the major glucose transporter GLUT-4 to the plasma membrane. In addition, activation of IRS-1 and 2 promotes the activation of the MAPK kinases ERK 1 and 2 that translocate into the nucleus and regulate transcriptional processes. White Mulberry extracts (Flavonoids, Polysaccharides, 1-DNJ) exert their action on glucose homeostasis by potentiating the IRS-1 and IRS-2-dependent pathways, and by activation of the AMP-activated protein kinase, which, in turn, potentiates the translocation of GLUT-4 from the cytosol to the plasma membrane. AMPK: AMP-activated protein kinase, P: Phosphorylation, Tyr: Tyrosine.

Different studies have revealed the antidiabetic effects of White Mulberry leaves and fruits in rodent models of diabetes. Postprandial hypoglycemic effects of the aqueous leaf extract and leaf powder of White Mulberry have been investigated using Goto-Kakizaki (GK) and Wistar rats [4]. The effect of oral administration of leaf extract at 3.75 g·kg^−1^ on postprandial glucose responses was tested using maltose or glucose as a substrate. With maltose, the extract significantly reduced the peak of plasma glucose responses in both GK and Wistar rats. With glucose, the extract also significantly reduced blood glucose concentrations at 30 min in both animal models. These findings indicated that the leaf extract has a significant postprandial hypoglycemic effect, likely through the inhibition of α-glucosidase and glucose transport. Earlier studies also reported hypoglycemic activities in the leaves and root bark of White Mulberry. A single dose of aqueous extracts from the leaves and root bark at 200 mg·kg^−1^ decreased plasma glucose levels and enhanced the tissue glucose uptake [5]. Administration of mulberry root bark extract at 600 mg·kg^−1^·d^−1^ to STZ-induced diabetic rats for 10 days significantly reduced serum glucose and lipid peroxides while enhancing the insulin levels [39]. In a further study, two different doses of mulberry leaf extract, 400 and 600 mg·kg^−1^, were tested on STZ-induced diabetes. The study population consisted of five groups: (a) the control group, (b) the control group treated with mulberry leaf extract, (c) diabetic control group without treatment, (d) diabetic group treated with 400 mg mulberry leaf extract, and (e) diabetic group treated with 600 mg of mulberry leaf extract. Plasma glucose levels and other parameters, which were raised in the diabetic group, were restored to control levels with treatment at 600 mg·kg^−1^. The diameter of the islets and the number of β-cells, which had decreased in the diabetic group, were also restored to control levels after the treatment. This study documented that mulberry leaf extract at a dose of 600 mg·kg^−1^ has therapeutic effects in STZ-induced diabetes and can restore the reduced number of β-cells [40]. The antidiabetic effects of White Mulberry were further confirmed in different animal models of diabetes. In particular, in type II diabetic rats, treatment with 250 or 750 mg·kg^−1^ of the aqueous ethanol leaf extract of White Mulberry resulted in a decrease in plasma glucose levels [41]. In this case, the antidiabetic properties of the extracts were attributed to chlorogenic acid and rutin present in the extracts. Antidiabetic effects were also documented in Zucker diabetic fatty rats; in particular, these rats were fed with mulberry fruit extract at doses of 125 or 250 mg·kg^−1^ twice daily for five weeks. The treatment determined a significant reduction in glucose levels in comparison with the control group [42]. The extracts at a dose of 250 mg·kg^−1^ did not influence the histology of pancreatic β-cells, as well as the insulin levels. Another study documented in STZ-induced diabetic mice that flavonoids in the ethanol fruit extract (100 and 200 mg·kg^−1^) significantly decreased serum glucose and serum protein levels and increased antioxidant enzymatic activities [43]. The relevant α-glucosidase inhibition documented in the extract may partially explain the antidiabetic activity. More recent in vitro and in vivo experiments have suggested that White Mulberry leaf extracts and their components (1-DNJ, flavonoids, polysaccharides) can improve glucose metabolism through activation of the IRS-1/PI3K/Akt pathway and increase in GLUT4 expression and translocation, leading to improved glucose tolerance, insulin resistance, and control of body weight [44,45,46,47,48,49]. Moreover, Meng [49] showed that flavonoids from White Mulberry extract activated AMP-activated protein kinase (AMPK), a key molecule in glucose homeostasis (46). AMPK activation enhanced peroxisome proliferator-activated receptor γ co-activator 1-α (PGC-1α), increasing the mitochondrial function, antioxidant capacity, and GLUT4 expression and translocation. Figure 3 shows the principal molecular pathways involved in the improvement of glucose homeostasis induced by White Mulberry extracts.

### 9.2. Anti-Hyperlipidemia Effects

Mulberroside A, prepared from the ethanol root extract of White Mulberry, and its aglycone derivative, oxyresveratrol, produced by enzymatic conversion of mulberroside A, have been studied for their anti-hyperlipidemic effects in vivo, by using two different rodent models [50]. Oral pre-treatment with mulberroside A or oxyresveratrol (1–5 mg·kg^−1^) decreased plasma levels of lipids in hyperlipidemic rats and in rats fed with a hypercholesterolemic diet. Moreover, oxyresveratrol demonstrated a more pronounced lipid-lowering action compared with mulberroside A. These experimental data confirm the notion of hypolipidemic effects of the root bark [51] and hypotriglyceridemic effects of the leaves [52] of mulberry.

### 9.3. Anti-Atherosclerosis Effects

Several studies have documented that the leaves and fruits of White Mulberry have anti-atherosclerotic effects in rodents. In apolipoprotein E-deficient mice, an animal model of atherosclerosis, it has been reported that 12 weeks of treatment with a dietary intake of 1% mulberry leaf powder determined a significant delay in the appearance of oxidized lipoproteins, as well as a reduction in aortic atherosclerotic plaques by 40%. All together, these findings allow us to speculate that mulberry leaves have antioxidative properties that strongly scavenge free radicals and interfere with lipoprotein oxidation, helping to prevent the development of atherosclerotic lesions [53]. In a further study performed on New Zealand white rabbits fed with normal or high-cholesterol diet (HCD), and treated with or without either aqueous mulberry fruit extract at concentration of 0.5% or 1.0% for 10 weeks the plasma levels of cholesterol, triglycerides, and low-density lipoprotein cholesterol of HCD rabbits treated with the fruit extract resulted to be lower than those of the control group [54]. In the aortas of rabbits receiving 0.5% or 1.0% of the extract, a reduction in atherosclerotic lesions by 42–63% was documented compared with the controls. In another study, in rats fed with a high-fat diet and treated with freeze-dried mulberry fruit powder (5% and 10%), it was documented that the treatment decreased plasma levels and liver cholesterol and triglyceride content, as well as affected lipid peroxidation, and enhanced antioxidant enzyme activity, thereby inhibiting the development of atherosclerosis [36].

### 9.4. Anti-Obesity Effects

White Mulberry and its components have been found to significantly increase lipid degradation and to prevent body weight gain in an animal model of obesity. The effects of the ethanol leaf extract of White Mulberry on the activity of melanin-concentrating hormone, which regulates food intake at the central level, were studied in diet-induced obese mice [55]. The results from the hormone receptor assay show that the extract (10−100 μg·mL^−1^) had a relevant inhibitory effect, with an IC50 value of 2.3 μg·mL^−1^. Furthermore, the administration of 100, 250, and 500 μg·mL^−1^ for 32 days determined a reduction in body weight and peripheral and hepatic lipid accumulation. Consistently, in male hamsters fed a high-fat diet, the treatment with aqueous mulberry leaf extract resulted in a reduction in body weight and reduction in plasma levels of cholesterol, triacylglycerol, and free fatty acid concentrations, as well as an increase in the ratio HDL/LDL [56]. Another study [57] conducted on obese mice fed with a combination of leaf and fruit extract of White Mulberry at 500 mg·kg^−1^ for 12 weeks documented an increase in levels of cholesterol transfer proteins in association with a decreased oxidative stress, allowing the speculation that White Mulberry and its components are able to regulate lipid accumulation and prevent body weight gain in animal models of obesity.

The capability of mulberry to regulate the expression of genes involved in cholesterol and free fatty acid synthesis plays a pivotal role in its antilipidemic actions. For instance, it has been reported that White Mulberry fruit extract treatment in HCD-fed mice significantly decreased total cholesterol and triglycerides [58]. Furthermore, in vitro experiments in HepG2 cells showed that this effect was mediated by the enhanced expression of the low-density lipoprotein receptor and by the downregulation of the following enzymes: 3-hydroxy-3-methylglutaryl-coenzyme A (HMG-CoA) reductase, FAS, glycerol-3-phosphate acyltransferase (GPAT), and sterol regulatory element-binding protein 1 (SREBP1) [56]. Similar properties were confirmed in other studies, corroborating the ability of mulberry extracts to regulate lipid accumulation [59,60,61]. Moreover, the ethanolic extract of White Mulberry leaves (100 mg/kg body weight) was administered for 8 weeks to female Wistar rats fed with HCD in order to assess whether or not White Mulberry leaves could affect the development of obesity, dyslipidemia, insulin resistance, mood, and cognitive function. The same study also investigated the motor deficits associated with the secretion of adipokines from visceral adipose tissue. In animals fed with HCD, oral administration of White Mulberry compared with untreated rats decreased body weight gain, hypercholesterolemia, hypertriglyceridemia, atherogenic and coronary artery indexes, plasma glucose levels, and insulin resistance index. More interestingly, White Mulberry administration significantly reduced, in visceral adipose tissue, both serum leptin and resistin contents, as well as their mRNA expression, while significantly increasing serum adiponectin levels and its mRNA expression. In terms of behavioral alterations, White Mulberry reduced motor deficits, memory impairment, depression, and anxiety-like behavior [62]. All together, these results show that White Mulberry supplementation is able to reduce adiposity, insulin resistance, and behavioral deficits through the down-regulation of the visceral adipose tissue of leptin and resistin gene expression and simultaneous upregulation of adiponectin gene expression. Finally, a report by Noh et al. investigated the effects of a mulberry fruit ethanol extract on dyslipidemia, liver steatosis, and adipokine imbalance induced by a high-fat diet. Male Sprague Dawley rats were assigned to one of four groups: (A) control diet, (B) high-fat diet, (C) high-fat diet with mulberry fruit ethanol extract at 150 mg/kg/day, (D) high-fat diet with mulberry fruit ethanol extract at 300 mg/kg/day [63]. The study measured triglyceride, total cholesterol, high-density lipoprotein cholesterol, alanine aminotransferase, and aspartate aminotransferase activities. Leptin, adiponectin, and plasminogen activator inhibitor-1 (PAI-1) levels were also assessed. Plasma cholesterol and low-density lipoprotein cholesterol (LDL-C) levels, along with the triglyceride/high-density lipoprotein cholesterol ratio, increased in the high-fat diet group compared with the control group, whereas these values decreased in the high-fat diet with mulberry fruit ethanol extract at 150 mg/kg/day group (*p* < 0.05), indicating that mulberry fruit ethanol extract had a plasma lipid-lowering effect. However, mulberry fruit ethanol extract MBEE did not affect the high-density lipoprotein cholesterol level. Moreover, mulberry fruit ethanol extract administration markedly reduced hepatic triglyceride. The high-fat diet group had higher PAI-1 levels, and mulberry fruit ethanol extract treatment significantly reduced leptin levels, leptin/adiponectin, and PAI-1/adiponectin ratios. These findings suggest that mulberry fruit ethanol extract improved the imbalance between pro- and anti-inflammatory adipokines, shifting toward a more anti-inflammatory state. Therefore, mulberry fruit ethanol extract could protect against abnormal lipid metabolism and hepatic steatosis induced by a high-fat diet, through the reduction in plasma cholesterol and LDL-C levels, the improvement of the ratio between triglyceride/high-density lipoprotein cholesterol, and the reduction in the accumulation of hepatic triglyceride. These effects are associated with the regulation of leptin/adiponectin and PAI-1/adiponectin ratios.

### 9.5. Hepatoprotective Effects

The hepato-protective action has been described with leaves of White Mulberry. The hydroalcoholic leaf extract shows a significant protective effect on the liver in mice with carbon tetrachloride (CCl_4_)-mediated liver fibrosis [64]. Compared with the control, the plasma levels of aspartate aminotransferase and alanine aminotransferase were lower, sleep duration was shorter, and no signs of liver fibrosis or inflammation were found in CCl_4_ mice treated with the extract. The protective actions of aqueous mulberry fruit extract were confirmed in male Wistar rats with CCl_4_-induced liver fibrosis [65]. In these animals, oral administration of different doses (0.5%, 1%, and 2%) of the extract reduced peroxidation of lipids and prevented fat build-up and liver fibrosis. The extract also reduced the expression of pro-inflammatory genes like cyclooxygenase-2 (COX-2), nuclear factor kappa B (NF-κB), and inducible nitric oxide synthase (iNOS). These results suggest that the extract has both protective and healing effects against liver injury and fibrosis by lowering lipid peroxidation and stopping the activation of inflammation-related genes.

**Figure 4:**
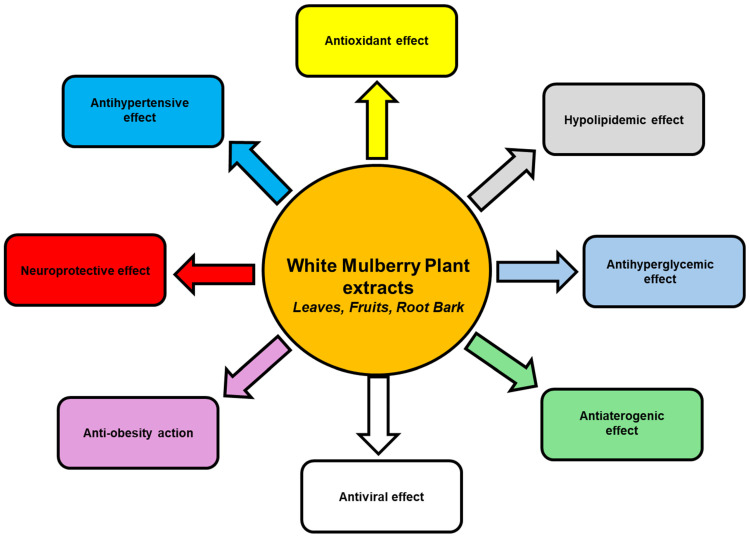
Principal pharmacological actions of White Mulberry that account for cardiovascular prevention.

In Figure 4, the principal pharmacological actions of White Mulberry that account for the reduction in cardiovascular risk are reported.

## 10. Clinical Trials

### 10.1. Hypoglycemic Effects

The effects of mulberry leaf extract on postprandial blood glucose levels were investigated in healthy and diabetic subjects. For this purpose, 1 g of mulberry leaf extract or placebo was administered after the ingestion of 75 g of sucrose dissolved in 500 mL of hot water. The study involved 10 healthy subjects and 10 type-2 diabetic (T2D) subjects who were receiving oral antidiabetic agents [66]. The results demonstrate that co-ingestion of mulberry extract significantly reduced blood glucose increments over the first 120 min compared with placebo. In particular, in healthy subjects, the increase in plasma glucose levels was significantly reduced in the mulberry group (15 ± 18 mg/dL) compared with the placebo group (22 ± 33 mg/dL; *p* = 0.005). In type-2 diabetic subjects, the increase was 42 ± 28 mg/dL with mulberry compared with 54 ± 46 mg/dL with placebo (*p* = 0.002). The placebo group exhibited greater declines in glucose levels below fasting at the end of the study, suggesting that the hypoglycemic effect of mulberry is likely due to inhibition of intestinal sucrase activity [67]. In another study, the effects of White Mulberry leaf extract on postprandial glucose and insulin levels were evaluated in patients with T2D who were being treated with sulfonylurea. Ten patients with T2D and 10 healthy subjects participated. After ingestion of jelly containing 3.3 g of mulberry leaf extract, the postprandial blood glucose and insulin levels in the diabetic group were significantly lower compared with placebo. In healthy subjects, the increment of serum glucose after the ingestion of the extract jelly was 97 mg/dL, compared with 125 mg/dL detected after ingestion of the placebo [68]. The effects of 1-DNJ have been investigated for its ability to suppress postprandial blood glucose levels. A study with food-grade mulberry powder enriched with 1.5% DNJ evaluated the response to sucrose load in healthy volunteers. The results show that doses of 0.8 and 1.2 g of DNJ-enriched powder significantly decreased postprandial plasma glucose and insulin synthesis, highlighting the potential of DNJ-enriched mulberry powder as a dietary supplement for the management of T2D [69]. A meta-analysis by Phirman pooled data from six studies to assess the effect of White Mulberry on blood glucose homeostasis [70]. The analysis showed that the reduction in fasting plasma glucose did not reach statistical significance [71,72,73,74]. Four randomized controlled trials were included in the meta-analysis for glycated hemoglobin (HbA1c). They showed that treatment with White Mulberry did not significantly reduce the values of HbA1C compared with the control (MD −0.05 mmol/L; 95% CI −0.19, 0.09; *p* = 0.49). Moreover, the pooled analysis showed that postprandial plasma glucose (PPG) levels at 30, 60, and 90 min were significantly lower in participants treated with White Mulberry compared with the controls. In particular, at 30 min, the mean difference in PPG levels was −1.04 mmol/L (*p* < 0.00001), at 60 min it was −0.87 mmol/L (*p* < 0.0001), and at 90 min it was −0.55 mmol/L (*p* = 0.001). However, no significant difference in PPG was observed at 120 min (*p* = 0.85). Furthermore, the pooled analysis revealed a trend toward a reduction in homeostasis model–insulin resistance index (HOMA-IR) levels, suggesting that White Mulberry may improve insulin sensitivity. After excluding a study where both White Mulberry and Korean ginseng were administered, a significant reduction in HOMA-IR was observed (MD −0.49; 95% CI −0.89, −0.08; *p* = 0.02). Phirman and colleagues hypothesized that the effects of White Mulberry on glucose homeostasis could be ascribed to its DNJ content [70]. DNJ has been shown to inhibit α-glucosidase activity [69,75], and higher concentrations of DNJ co-administered with White Mulberry were associated with a significant reduction in PPG levels, although the differences were not statistically significant due to the limited number of trials [69,71,76]. Despite these limitations, the overall available data indicate that White Mulberry may be beneficial in managing postprandial hyperglycemia and preventing the progression of diabetes and its complications [30,77]. Elevated peak of serum glucose during postprandial periods triggers an oxidative stress resulting in the progression of diabetes [78]. The potential effect of White Mulberry on PPG reduction leads to a decrease in oxidative stress and, therefore, possibly delays diabetic progression and the associated complications. However, there is no clinical study on the reduction in PPG by White Mulberry associated with clinical efficacy and reduction in complications. A study investigated the effects of White Mulberry extract on hepatic enzymatic activity, oxidative stress, insulin metabolism, lipid profiles, and markers of inflammation in patients with T2D. This randomized, double-blind, placebo-controlled study recruited 60 patients, who were randomized into two study groups: one group took 300 mg of White Mulberry extract twice daily (*n* = 30), while the other received a placebo (*n* = 30). Fasting blood samples were collected at baseline and after 12 weeks of treatment to measure related markers. The results show that White Mulberry extract significantly reduced insulin levels (*p* < 0.026) and malondialdehyde (MDA) (*p* < 0.001), and significantly increased HDL-cholesterol levels (*p* < 0.001) compared with the placebo. However, the extract did not have an impact on other metabolic profiles. These findings indicated that White Mulberry extract improves insulin, HDL-cholesterol, and malondialdehyde levels in patients with T2D, but does not affect other metabolic parameters [79].

### 10.2. Hypolipidemic Effects

The hypolipidemic effects of mulberry leaf powder were compared with the effects of glibenclamide, a standard antidiabetic drug [80]. This study involved 24 patients with T2D, who were divided into two groups. One group received mulberry leaf powder (3 g/day) for 30 days, while the other group took 5 mg of glibenclamide per day for the same period. Serum and erythrocyte membrane lipid profiles were analyzed in basal conditions and at the end of the treatment. The results show that mulberry leaf powder significantly reduced plasma levels of cholesterol (12%), triglycerides (16%), plasma free fatty acids (12%), LDL-C (23%), very-low-density lipoprotein cholesterol (17%), plasma peroxides (25%), and urinary peroxides (55%). Additionally, it significantly increased high-density lipoprotein cholesterol by 18%. On the contrary, the glibenclamide group showed only small changes in lipid profiles, with reductions in triglycerides (10%), plasma peroxides (15%), and urinary peroxides (19%), but no significant changes in other lipid markers. These results suggest that mulberry leaf powder has stronger effects on lipid profiles and oxidative stress markers than glibenclamide. A further study investigated the DNJ-enriched mulberry leaf extract on plasma lipid profiles. Ten male subjects with serum triglyceride levels ≥200 mg/dL were treated with 12 mg of DNJ-rich mulberry leaf extract three times a day before meals for 12 weeks. Although the mean serum triglyceride levels decreased from 312 ± 90 mg/dL at baseline to 269 ± 66 mg/dL and 252 ± 78 mg/dL at week 6 and week 12, respectively, these changes did not result in statistically significant changes. Furthermore, there were no major changes observed in total cholesterol, LDL-cholesterol, or high-density lipoprotein cholesterol [81]. A further clinical study assessed the hypolipidemic effects of mulberry leaf in patients with mild dyslipidemia [82]. Treatment with 280 mg of mulberry leaf powder and 0.37 mg of DNJ, administered three times daily for 12 weeks. At weeks 4 and 8, serum triglyceride levels significantly decreased by 10% and 13%, respectively. At the end of the study, total cholesterol, triglyceride, and LDL-C levels decreased by 4.9%, 14%, and 5.6%, respectively, while high-density lipoprotein cholesterol increased by 20%. The meta-analysis by Phirman et al. [70] found that White Mulberry treatment did not lead to statistically significant differences in plasma levels of LDL-C (MD −0.11; 95% CI −0.52, 0.30; *p* = 0.61), total cholesterol (MD −0.17; 95% CI −0.62, 0.27; *p* = 0.45), high-density lipoprotein cholesterol (MD 0.00; 95% CI −0.10, 0.11; *p* = 0.94), or triglycerides (MD −0.20; 95% CI −0.42, 0.02; *p* = 0.08) when compared with the control group. Considering the cholesterol-lowering effects, it may be useful to explore combining White Mulberry with other principles to enhance its effectiveness. By integrating multiple components, a broader range of mechanisms may be activated to improve lipid profiles, potentially overcoming the issue of a small percentage of subjects reaching the desired therapeutic targets. At this regard, a randomized, cross-over, double-blind study [72] compared, over a 4-week treatment period, two formulations approved in Italy for the management of dyslipidemia (LopiGLIK and Armolipid Plus), both containing Monacolin K and Berberine but differing in that LopiGLIK includes White Mulberry, while Armolipid Plus contains Policosanol, Astaxanthin, Folic acid, and Coenzyme Q10. The study confirmed that both formulations were effective in improving the lipid profile in patients with hypercholesterolemia who either did not require statins or were intolerant to them [83,84]. However, the addition of White Mulberry extract, due to its alpha-glucosidase inhibitory and antioxidant properties, enhanced the cholesterol-lowering effect of the combination. Notably, likely due to a more comprehensive action on plasma lipids, this combination was able to normalize plasma LDL-C levels in approximately 50% of the study population. This higher percentage of positive therapeutic responses resulted in a more pronounced reduction in average plasma total and LDL-C concentrations compared with the other formulation. When glucose metabolism parameters were evaluated, a significant decrease in fasting glucose, insulin, and HbA1c levels was observed only in the group treated with the combination containing White Mulberry. Consequently, there was also a significant difference between these values and those measured at the end of the follow-up period in the other treatment group. No patient experienced clinically evident hypoglycemia. The HOMA index, calculated according to the formula by Matthews [85], used to assess the impact of both treatments on insulin resistance, showed that only the White Mulberry combination led to an improvement in insulin sensitivity. Therefore, the findings of this study further support the evidence that White Mulberry improves glucose metabolism [43] and, consequently, that in combination with red yeast rice and berberine, it may contribute to reducing global cardiovascular risk through mechanisms beyond cholesterol lowering. Since a continuous relationship between HbA1c levels and cardiovascular disease, as well as all-cause mortality, has been demonstrated in men and women without diabetes, HbA1c can be used to assess whether the combination, including White Mulberry, affects glucose homeostasis and may potentially improve cardiovascular outcomes. A reduction in HbA1c was recorded, which was statistically significant though modest in extent. However, it is well recognized that HbA1c is a reliable marker of glycemic control over the previous three months [86,87]. Thus, even a slight reduction in HbA1c after only four weeks of treatment could represent a clinically meaningful change in glucose metabolism. The concurrent reductions in fasting glucose and insulin levels are consistent with the HbA1c decrease and may be attributed to improved insulin sensitivity observed during treatment with the White Mulberry combinations. Nonetheless, although the study demonstrated a significant improvement in both lipid and glycemic profiles, the small sample size necessitates confirmation in larger populations. Additionally, the short duration of follow-up limits the ability to generalize the findings on HbA1c reduction, which requires longer observation to fully evaluate the trend. To address these limitations, a subsequent study was carried out to verify whether the combination containing White Mulberry (Berberine 531 mg, Red yeast rice powder 220 mg: 3.3 mg Monacolin K, and White Mulberry 200 mg) could be more effective in improving lipid and glucose metabolism compared with an already established nutraceutical combination used for the same therapeutic purpose (Policosanol 10 mg, Red yeast rice 200 mg: 3 mg Monacolin K, Berberine 500 mg, Astaxanthin 0.5 mg, Folic acid 200 mcg, and Coenzyme Q10 2 mg) [88]. Thirty-one physicians participated in this multicenter, randomized, controlled, single-blind trial, each asked to enroll the first 25 consecutive adult outpatients. Inclusion criteria were: (A) age between 18 and 75 years, (B) mild hypercholesterolemia or metabolic syndrome not requiring statin therapy or with statin intolerance; while, exclusion criteria included (A) pregnancy or breastfeeding, (B) current use of lipid-lowering, blood-pressure-lowering, or glucose-lowering drugs, (C) a previous history of cardiovascular disease, (D) any neurological or psychiatric disorder that could, at least in part, interfere with the ability to provide informed consent. Each study site gathered data during the first half of 2016. At the end of the 16-week follow-up, 209 out of the 359 enrolled patients (58%) reached the primary endpoint (LDL-C levels below 130 mg/dL), with 72.0% in the White Mulberry combination group and 43.0% in the comparator group (*p* < 0.0001). Based on these results, a true difference in cardiovascular risk of at least 29 percentage points between the two groups was demonstrated, which was statistically significant with more than 99% power (Z value for *p* < 0.05, two-tailed). Baseline clinical characteristics and metabolic profiles did not differ significantly between the two groups. At follow-up, body weight had decreased in both groups compared with baseline, but the reduction in the White Mulberry group was significantly greater. Moreover, in the White Mulberry group, weight changes showed a significant correlation with plasma triglyceride levels and an inverse correlation with systolic blood pressure. In contrast, in the alternative treatment group, changes in weight correlated more closely with variations in HOMA index and systolic blood pressure. Both treatments significantly reduced LDL-C levels at the end of 16 weeks (*Morus alba* group: −35.2 ± 20.8 mg/dL; alternative group: −24.7 ± 22.8 mg/dL; *p* < 0.0001 for both comparisons). There was also a significantly greater percentage reduction in LDL-C levels in the White Mulberry group (−21.9 ± 12.1%) compared with the alternative group (−15.1 ± 13.9%; *p* < 0.0001). Total cholesterol levels were also significantly reduced in both groups, but more prominently in the White Mulberry group (−17.7 ± 8.1% vs. −11.6 ± 9.8%; *p* < 0.0001), with a similar advantage observed for triglyceride reductions. After 16 weeks, high-density lipoprotein cholesterol concentrations did not change significantly in either group. Both treatments lowered fasting plasma glucose, HbA1c, plasma insulin, and HOMA index values, but when comparing the two groups, statistically significant differences emerged in all parameters except insulin, consistently showing a more pronounced effect with the White Mulberry combination. The magnitude of the difference between the two treatments cannot be explained by the small dosage increases in Berberine (531 mg vs. 500 mg) and Monacolin K (3.3 mg vs. 3 mg) in the White Mulberry combination, since it has been shown that reducing Monacolin K to 3 mg does not affect the lipid-lowering action of this formulation [89]. Furthermore, a study conducted in postmenopausal women demonstrated that a nutraceutical formula containing a comparable dose of White Mulberry induced a similar LDL-C reduction [90]. These observations support the hypothesis that White Mulberry itself contributes to enhanced cholesterol reduction and improved glycemic control, possibly by acting through additional pathways beyond those of Monacolin K and Berberine. This may also explain the higher percentage of patients achieving LDL-C levels below 130 mg/dL. More recently, the Mulberry extracts were tested in obese individuals to evaluate their effects on cardiovascular risk factors. In a randomized, single-blind crossover study, it was documented that the concentrated mulberry drink containing 1041.90 mg of phenolic compounds and 35.34 mg of anthocyanins reduced both systolic and diastolic blood pressure, without any effect on the serum levels of total cholesterol, LDL-C, and HDL-C, while decreasing the levels of triglycerides. Moreover, the drink reduced the plasma concentration of the C-reactive protein [91]. The effects of supplementation of leaf extracts of White Mulberry on the postprandial glucose and insulin changes have been further investigated in a randomized, crossover, single-blinded clinical study. In this study, 166 healthy subjects were treated with a combination of aqueous extracts of White Mulberry and the rind of *Malus Domestica* or a placebo. Postprandial blood glucose and insulin levels were measured after a carbohydrate-rich meal or sucrose drink. The combination of White Mulberry and Malus Domestica reduced the meal-evoked increase in the area under the curve of glucose and insulin [92]. This indicates that this combination is able to modulate the postprandial glucose homeostasis. The results of principal clinical studies of White Mulberry on glucose and lipidic metabolism are summarized in Table 1.

## 11. Future Perspectives

In the last decades, a significant increase in metabolic diseases has been recorded, such as type 2 diabetes, metabolic syndrome, and obesity [93]. Among the different therapeutic effects of the White Mulberry extracts, its ability to modulate glucose homeostasis and weight loss has been clearly documented [11,12]. The pre-diabetes and overweight, although they cannot be considered diseases, are conditions associated with an increased susceptibility for the development of type 2 diabetes mellitus and subsequent cardiovascular diseases. Therefore, they are the perfect target for a real primary prevention intervention. Although the clinical data that document the beneficial effects of treatment with White Mulberry extracts to prevent the development of diabetes are very promising, they lack a large sample size and long follow-up. Therefore, in the future, clinical studies are required to confirm the beneficial effects of White Mulberry in the larger population with longer follow-up.

## Figures and Tables

**Table 1:** Summary of the results of the principal trials that document the effects of White Mulberry on glucose and lipids.

Study Design	Individuals	Dose	Outcome	References
Randomized crossover study	10 healthy + 10 type 2 diabetic patients	1 g of Mulberry Leaf Extract or placebo	↓ blood glucose level after sucrose tolerance test	[66]
Single blinded, placebo-controlled study	10 healthy and 10 type 2 diabetes patients treated with or without sulfonylurea	3.3 g of mulberry leaf extract	↓ postprandial blood glucose level ↓ insulin	[68]
Randomized controlled study	24 healthy	6, 12, and 18 mg of 1-DNJ, or placebo	↓ postprandial blood glucose level ↓ insulin after sucrose tolerance test	[69]
Randomized double-blind cross over study	12 individuals with impaired glucose tolerance	Mulberry leaf extract capsule(s) containing 3, 6 and 9 mg of 1-DNJ or placebo	↓ postprandial glycemic control = glycated hemoglobin	[71]
Randomized double blind cross-over study	23 patients with hypercholesterolemia	Policosanol (10 mg), Red yeast rice (200 mg; 3 mg monacolin K), Berberine (500 mg), Astaxantine (0.5 mg), Folic Acid (200 mcg) and Coenzyme Q10 (2 mg) vs. Berberine (531.25 mg), Red yeast rice powder (220 mg; 3.3 mg monacolin K) and leaf extract of *Morus alba* (200 mg).	↓ fasting plasma glucose ↓ fasting insulin ↓ HOMA index ↓ HbA1c	[72]
Randomized, placebo-controlled, double-blind, parallel study	94 individuals with impaired glucose tolerance or mild type 2 diabetes	Mixture of ginseng roots, mulberry leaf water extract, and banaba leaf water extract or placebo	= fasting glucose = fasting insulin = HOMA-IR ↓ ICAM-1 ↓ VCAM-1 ↓ ox-LDL	[73]
Randomized, double-blind, placebo-controlled trial	50 healthy	4.5, 9, 18 mg of 1-DNJ or placebo	↓ postprandial glucose during maltose tolerance test	[76]
Randomized, double-blind, placebo-controlled trial	60 patients with type 2 diabetes.	White Mulberry extract (300 mg) or placebo twice a day.	↓ insulin ↑ HDL-C ↓ MDA	[79]
Open-label, single-group study	10 subjects with hypertriglyceridemia	36 mg of 1-DNJ	= TG = LDL-C = HDL-C	[81]
Open-label, single-group study	23 subjects with mild dyslipidemia	280 mg mulberry leaf three times a day	↓ TG ↓ LDL-C ↑ HDL-C ↓ cholesterol	[82]
Randomized, single-blind trial	359 subjects with mild hypercholesterolemia	Policosanol (10 mg), Red yeast rice (200 mg; 3 mg monacolin K), Berberine (500 mg), Astaxantine (0.5 mg), Folic Acid (200 mcg) and Coenzyme Q10 (2 mg) vs. Berberine (531.25 mg), Red yeast rice powder (220 mg; 3.3 mg monacolin K) and leaf extract of *Morus alba* (200 mg).	↓ TG ↓ LDL-C ↓ cholesterol ↓ systolic blood pressure ↓ diastolic blood pressure ↓ fasting insulin ↓ HOMA-IR	[88]
Single-blind, randomized crossover study	12 obese subjects	100 g of concentrated mulberry drink or placebo	↓ TG = LDL-C = HDL C = cholesterol ↓ systolic blood pressure ↓ diastolic blood pressure ↓ C-reactive protein	[91]
Randomized, crossover, single-blinded clinical study	166 healthy	500 mg of aqueous extracts of White Mulberry and rind of Malus domestica, or placebo	↓ area under the curve of postprandial glucose ↓ area under the curve of postprandial insulin	[92]

↓ Decrease; ↑ Increse; = Unchanged.

## Abbreviations

AMPK	AMP-activated protein kinase
CCl_4_	Carbon tetrachloride
HbA1c	Glycated hemoglobin
GPAT	Glycerol-3-phosphate acyltransferase
HDL-C	High-density lipoprotein cholesterol
HCD	High-cholesterol diet
HOMA-IR	Homeostasis model–insulin resistance index
ICAM-1	Intracellular adhesion molecule-1
LDL-C	Low-density lipoprotein cholesterol
MDA	Malondialdehyde
ox-LDL	Oxidized low-density lipoprotein
PAI-1	Plasminogen activator inhibitor-1
PPG	Postprandial plasma glucose
TG	Serum triglyceride
STZ	Streptozotocin
SREBP1	Sterol regulatory element-binding protein 1
T2D	Type-2 diabetic
VCAM-1	Vascular cell adhesion molecule-1
1-DNJ	1-deoxynojirimycin
HMG-CoA	3-hydroxy-3-methylglutaryl-Coenzyme A
